# Clinical experience of debridement combined with resorbable bone graft substitute mixed with antibiotic in the treatment for infants with osteomyelitis

**DOI:** 10.1186/s13018-018-0916-9

**Published:** 2018-08-30

**Authors:** Zhiqiang Zhang, Hao Li, Hai Li, Qing Fan, Xuan Yang, Pinquan Shen, Ting Chen, Qixun Cai, Jing Zhang, Ziming Zhang

**Affiliations:** Department of Pediatric Orthopedics, Xinhua Hospital, School of Medicine, Shanghai Jiao Tong University, 1665 Kongjiang Road, Yangpu District, Shanghai, 20092 China

**Keywords:** Infants, Osteomyelitis, Pyogenic arthritis, Debridement, Resorbable bone graft substitute, Antibiotics

## Abstract

**Background:**

Osteomyelitis (OM) is an uncommon disease that originates from many different mechanisms in children. Treatment often involves a combination of surgical debridement combined and antibiotic therapy. The purpose of this article is to evaluate the effect of debridement combined with a new resorbable bone graft substitute (RBGS) mixed with antibiotics in the treatment of infants with OM.

**Methods:**

Twenty-two patients diagnosed with OM at our institution underwent debridement combined with implantation of RBGS mixed with vancomycin within 48 h after admission. Clinical and epidemiological factors, preoperative and postoperative radiographs, and laboratory parameters, including white blood cell (WBC), C-reactive protein (CRP), erythrocyte sedimentation rate (ESR), and neutrophil percentage (NEU%), were documented. The function of the involved extremity was evaluated at the final follow-up.

**Results:**

The mean age was 6.3 ± 4.8 months (range, 0.5 to 12 months). The mean duration of the symptoms was 14.5 ± 8.4 days (range, 2 to 30 days). The average length of hospitalization was 13.7 ± 6.2 days (range, 6 to 28 days). 13.64% (3/22) had positive results of purulent material obtained at the time of open biopsy and 18.18% (4/22) had positive blood cultures. The most common sites were located in the proximal femur (12), the distal femur (3), and the proximal humerus (3). Ten patients presented with concurrent pyogenic arthritis, while another 12 infants suffered from simple isolated hematogenous OM. The mean follow-up time was 3.0 ± 1.6 years (range, 1.0 to 6.0 years). Seven of 22 patients (31.82%) had complications such as limb length deformity (LLD), avascular necrosis (AVN), and pathologic subluxation of the hip. Fifteen out of 22 (68.18%) patients achieved good results. Additionally, patients who had concomitant pyogenic arthritis were more likely to develop complications than those with isolated OM (*p* = 0.02).

**Conclusions:**

Early debridement combined with implantation of RBGS mixed with vancomycin in the treatment of infants with OM achieved acceptable results in this series. Compared to those with simple isolated OM, patients with secondary pyogenic arthritis had a more virulent course.

## Background

Osteomyelitis (OM) is postulated to occur as a result of bacteremia and local trauma and occurs in children because of the rich blood supply to the growing bones. The clinical features and severity may differ somewhat based on the causative organism, children’s age, immune level, and other factors [1]. The long bone metaphysis is the most vulnerable part. If the infection has not been effectively controlled or treated, the whole bone will become involved and may lead to bone defects [2]. Concomitant pyogenic arthritis in infants occurs in the hip, knee, and other large joints, mostly as the result of OM extended to the adjacent joints. Due to the presence of the transphyseal vessels, as well as immature immune system, an epiphyseal OM secondary to septic arthritis could occur in an infant, especially without a timely and effective treatment [3].

Despite intervention with antibiotic therapy, the treatment of OM is still challenging on account of delays in treatment, drug-resistant bacteria, and inadequate treatment. Administration of antibiotics by local delivery with “filling agents” can result in an increased local concentration of antibiotics at the infection site. This technique has been described as an option to treat some conditions. For example, calcium sulfate pellets impregnated with antibiotics were applied successfully in the treatment of chronic OM and non-union in adults [4, 5].

To our knowledge, there are no previous reports focusing on infants with OM treated with debridement combined with resorbable calcium sulfate mixed with antibiotics. Therefore, the objective of this study was to evaluate the outcome of early debridement combined with implantation of resorbable bone graft substitute (RBGS) mixed with antibiotics for the treatment of OM in infants.

## Methods

After the institution’s Ethics Committee approval, a retrospective review was performed and all patients with the diagnosis of OM between January 1, 2012, and December 31, 2016, at our institution were enrolled. The inclusion criteria were (1) infants younger than 1 year old; (2) diagnosed with OM, (3) patients who underwent debridement combined with RBGS mixed with antibiotics, and (4) surgery within 48 h after admission.

OM was defined as having symptoms for several days with clinical and radiological features. Contrary to common belief, the main clinical symptom in infants may be as subtle as local irritability (e.g., a child crying on changing of diapers or touching the affected extremity) [6]. Laboratory findings such as increased WBC, ESR, and CRP plus radiological investigations supplement the clinical symptoms. A diagnosis of concurrent septic arthritis was made in patients who were found to have purulent material in the joint capsule at the time of surgical treatment of the OM.

Clinical and laboratory data were documented during and after admission. Physical examination data points including increased body temperature, local swelling, and limited range of motion. Meanwhile, radiological and laboratory tests were performed preoperatively. Namely, plain radiographs, CT scan, or MRI were taken to determine the site and area of the lesion. WBC, ESR, and CRP were tested immediately after hospitalization. Blood samples and cultures of purulent material obtained on aspiration or at the time of open exploration, debridement, or arthrotomy were collected for bacteria culture and sensitivities of the causative organism.

Antibiotic treatment by intravenous or oral therapy was started after admission, and the options of antibiotics were guided by local microbiology advice wherever possible to facilitate a more focused therapeutic regimen.

Surgical intervention was warranted if the child had persistent pain and fever after 48 h or bone lesion identified by MRI or plain radiograph or if there was evidence of a collection of purulent material by physical exam or advanced imaging. Patients who were observed to have bone lesions were given the treatment of RBGS combined antibiotics. Other interventions such as multiple surgical debridements and the usage of vacuum sealing drainage (VSD) were more often used for severe septic arthritis in our institution. Cultures from bone or joint fluid samples were processed during the surgeries. Open biopsy was done in cases where other diagnostic entities were considered (i.e., sarcoma). Antibiotic susceptibility test was taken simultaneously to guide for adjustment of the antibiotic regime. The necrotic and infectious bone lesion was excised completely and flushed with hydrogen peroxide, chlorhexidine, or saline repeatedly. After irrigation and debridement, the bone defect was filled with RBGS (OSTEOSET resorbable bead kit, Wright Medical Group, NV.), which was mixed with 0.5 g vancomycin. To prepare the RBGS, all the diluent was added to both calcium sulfate and antibiotic powders in a mixing bowl and mixed to a “dough”-like consistency (mixed thoroughly for 30–45 s). After molded and dried on the template, the molded RGBS beads were filled into the bone defect as many as possible. The affected limb was immobilized by a cast or brace to prevent possible pathologic fracture.

Postoperatively, multidisciplinary management included pediatric surgery intensive care unit, orthopedics, microbiology/infectious disease consultants, radiology, and nursing. Based on the results of bacteria culture, the antibiotic therapy was adjusted. If the results were negative, the previous regime was continued. WBC, CRP, ESR, and NEU% were noted every 48 h postoperatively.

Complications were documented at the final follow-up. Good outcomes were defined as no complications and without recurrence until the latest follow-up. Fisher exact test was used to compare the rates of complication between both groups (OM concurrent with pyogenic arthritis group and isolated OM group). Outcomes are expressed with *p* value. A 5% level of significance was used in analyses (SPSS19.0, IBM, USA).

## Results

Thirty-four patients were treated for OM in our hospital between January 2012 to December 2016. Seven patients who had repeated surgeries, using Vacuum Sealing Drainage (VSD) rather than RBGS, were excluded in this series. There were five patients lost to follow-up. Finally, 22 infants were included in the present study (16 boys and 6 girls). The mean follow-up time was 3.0 ± 1.6 years (range, 1.0 to 6.0 years). The mean age was 6.3 ± 4.8 months (range, 0.5 to 12 months). The mean duration of the symptoms was 14.5 ± 8.4 days (range, 2 to 30 days). 13.6% (3/22) had positive surgical cultures, and 18.2% (4/22) had positive blood cultures. The average length of hospitalization was 13.7 ± 6.2 days (range, 6 to 28 days). The most common sites affected by infection were the proximal femur (12), the distal femur (3), and the proximal humerus (3). The right proximal fibula and the right proximal radius were involved in one case, respectively. Ten patients had OM concurrent with pyogenic arthritis, while 12 infants suffered from isolated OM (Table 1).

**Table 1:** Demographic data of enrolled patients

Number	Sex	Age(months)	Location	Septic arthritics	Pathogen	The last time follow-up	Follow-up time(years)
1	M	2	RDF	N	N	Good	2.0
2	M	12	LDF	N	N	LLD	5.7
3	F	1.5	LDF	N	*Staphylococcus aureus*	Good	6.0
4	F	2.5	RPF	N	*Staphylococcus aureus*	Good	5.7
5	M	1	LPH	N	N	Good	3.2
6	F	10	LPT	N	N	Good	4.9
7	M	12	RF	N	N	Good	2.0
8	M	0.4	RPH	N	N	Good	3.3
9	M	9	RPT	N	N	Good	1.1
10	M	7	RPF	N	Gram-positive cocci	Good	3.5
11	M	10	RPH	N	N	Good	3.5
12	M	8	RPF	N	N	Good	1.1
13	M	1	LPF	Y	*Acinetobacter baumannii*	PHD LLD	1.0
14	M	1	LPF	Y	*Staphylococcus aureus*	AVN	1.1
15	M	0.67	RPF,	Y	N	Good	4.3
16	F	1	LPF	Y	*Staphylococcus aureus*	LLD	2.7
17	M	11	LPF	Y	N	LLD,	1.5
18	M	11	LPF	Y	N	Good	4.5
19	F	12	RPR	Y	N	Good	1.8
20	F	11	LPF	Y	N	Good	3.3
21	M	2	LPF	Y	*Staphylococcus warneri*	AVN	1.9
22	M	12	RPF	Y	N	AVN	2.4

*F* female, *M* male, *Y* yes, *N* no, *RDF* right distal femur, *LDF* left distal femur, *RPF* right proximal femur, *LPF* left proximal femur, *LPH* left proximal humerus, *LPT* left proximal tibia, *RF* right fibula, *RPH* right proximal humerus, *RPT* right proximal tibia, *RPR* right proximal radius, *LLD* limb length discrepancy, *PHD* pathological hip dislocation, *AVN* avascular necrosis

**Fig. 1:**
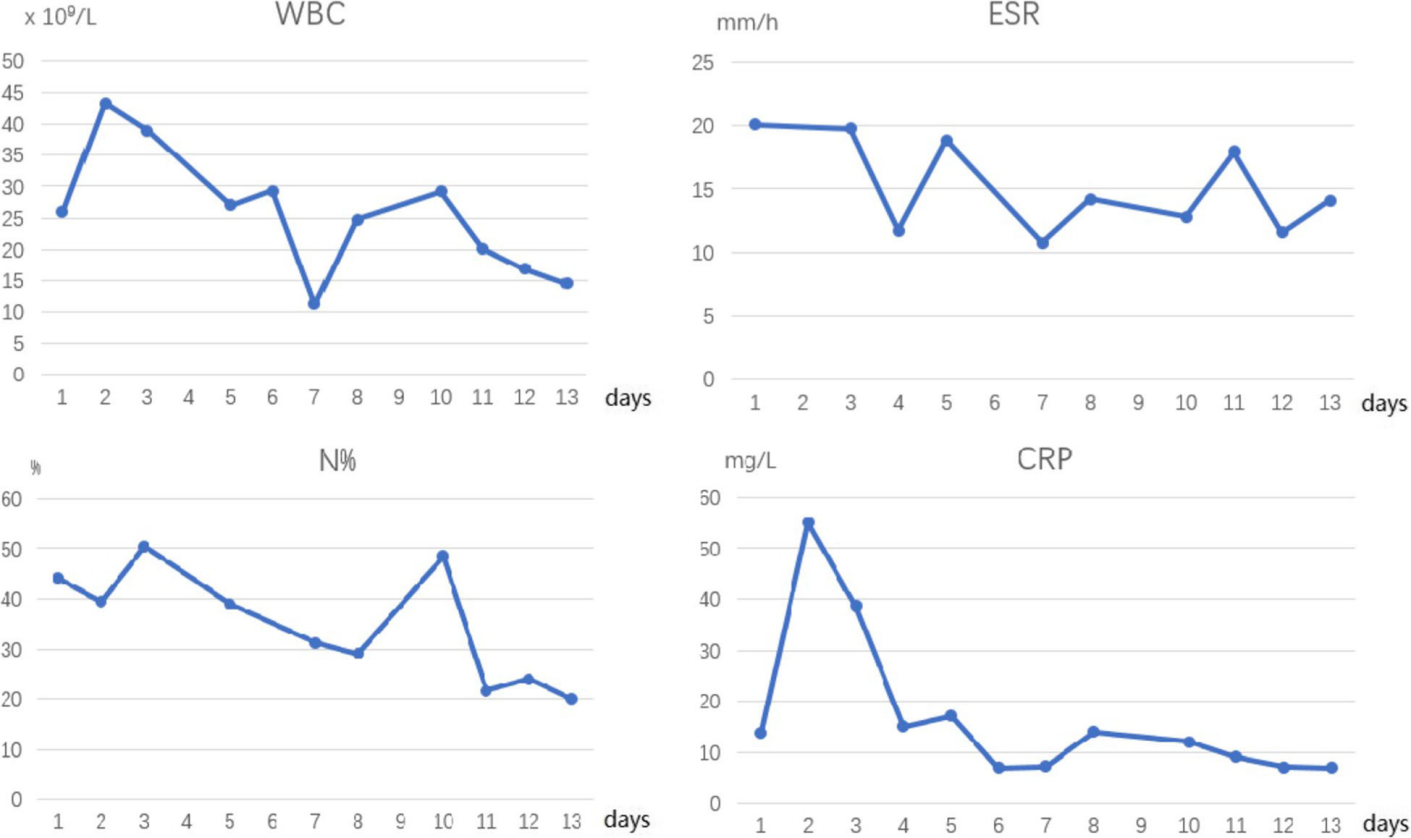
Changes of patients’ laboratory markers. The mean value of each laboratory marker consisted of those plots. WBC, white blood cell; ESR, erythrocyte sedimentation rate; NEU%, neutrophil%; CRP, C-reactive protein

All patients had a clinical examination and laboratory tests upon admission. The mean temperature on admission was 37.0 ± 0.4 °C. One patient (4.5%) had a temperature over 37.8 °C. All patients had decreased activity and cried on the passive movement of the extremity. Limb tenderness was present in 40.9% (9/22). The mean WBC was 14.1 ± 6.7 × 10 ^^ 9^/L (5.5–33.8 × 10 ^^ 9^/L), ESR was 33.5 ± 23.2 (2–85) mm/h, CRP was 15.5 ± 13.6 (7–55) mg/L (CRP was recorded as 7 when the result was less than 8.), and NEU% was 45.5 ± 19.5% (7.5.0–87.5%). In our study, children who undergo a surgical debridement are then serially monitored every 48 h with repeated inflammatory indices (WBC, ESR, and CRP) [7]. The tendency of WBC and CRP had risen on the first day after surgery, maybe due to the stress response. In the postoperative course, the trend of lab biomarkers was generally decreased. ESR and CRP had a similar tendency, which recovered within 6 days. WBC trend was slower, with a mean time of 2 weeks to return to normal range, whereas that of NEU% showed no trend (Fig. 1).

The most common complication was LLD, which was occurred in four patients by review of available imaging. Other complications included AVN of the femoral head in three patients and pathologic subluxation of the hip in one patient. At the final follow-up, 15 patients (68.2%) had no complications and were graded as good outcomes, while seven patients (31.8%) had complications.

There were no differences in complications occurrence between sexes (*P* = 0.62), temperatures ((*P* = 0.09), duration of symptoms (*P* = 0.93), WBC (*P* = 0.58), CRP (*P* = 0.97), NEU% (*P* = 0.71), ESR (*P* = 0.47), or the length of hospitalization (*P* = 0.29). However, patients who had concomitant pyogenic arthritis were more likely to develop complications than those with isolated osteomyelitis (*P* = 0.02). (Table 2).

**Table 2:** Comparison between cases with complications and those without complications

	Good outcomes	Complications	*P*
Cases, no.	15	7	
Sex, no.			0.62
F	5	1	
M	10	6	
Ages, average (range), months	6.5 ± 4.6 (0.5–12)	5.7 ± 5.6 (1–12)	0.73
Temperature, average (range), °C	36.9 ± 0.3 (36.5–37.5)	37.3 ± 0.5 (36.8–38.2)	0.09
WBC, average (range), × 10^9/L	13.4 ± 5.8 (5.5–28.2)	15.5 ± 8.7 (9.3–33.8)	0.58
CRP, average (range), mg/L	15.5 ± 14.1 (7–55)	15.3 ± 13.3 (7–38)	0.97
ESR, average (range), mm/h	30.7 ± 20.3 (2–70)	39.7 ± 29.2 (10–85)	0.47
NEU%, average (range), %	45.2 ± 23.9 (7.5–87.5)	43.7 ± 11.2 (28.1–62.1)	0.71
Duration of symptoms, average (range), days	14.4 ± 8.7 (2–30)	14.7 ± 8.3 (7–30)	0.93
Hospitalization, average (range), days	12.7 ± 5.0 (6–23)	15.7 ± 8.3 (7–28)	0.29
Septic arthritis, no.			0.02
Y	11	1	
N	4	6	

*F* female, *M* male, *WBC* white blood cell, *CRP* C-reactive protein, *ESR* erythrocyte sedimentation rate, *Y* yes, *N* no

## Discussion

The occurrence of OM is uncommon but missed or delayed diagnosis can lead to catastrophic consequences due to the unique vascular structure in infants. Because the transphyseal vessels persist until 15 to 18 months of age, infection in the metaphysis can spread into the epiphysis and produce concurrent septic arthritis [8–10]. Furthermore, joint effusion or empyema may be associated with joint dislocation and AVN [11]. Therefore, prompt diagnosis and early treatment are essential for good outcomes [12].

The diagnosis of OM might be difficult in infants because the clinical manifestations are not as evident as in older children and adults. In our study, one child (5.00%) had a temperature over 37.8 °C. All patients had decreased activity and cried on the passive movement of the extremity. Limb swelling and erythema were noted on the affected limb in 40.9% (9/22) of subjects. In comparison with patients from 1968 to 1972, Goergens [13] found that most patients suffering from OM and septic arthritis presented with only mild symptoms instead of the traditional symptoms of infection, such as fever, swelling, and clear decreased range of motion. In that study, the most common symptom was (> 90% of patients) refusal to move the affected limb, whereas only 50% of children showed local swelling and 32% children had no fever. Therefore, even if with normal body temperature and laboratory parameters, the diagnosis of OM should not be ruled out. MRI and bone scan were recommended to search for evidence of infection.

We quantified the biochemical markers in the recovery of postoperative course. CRP was found to be a better match for recovery, which was compatible with other reports [14]. Evidence has suggested that the CRP has a half-life of 19 h, rising and normalizing faster, and is easily tested, which makes it an ideal parameter for monitoring the improvement of pediatric infections [15]. Unkila-Kallio et al. [16] elaborated that the level of CRP usually decreased rapidly if the children have isolated OM. If it does not decrease, associated septic arthritis should be suspected. In our study, the trend of NEU% showed no trend, which was less meaningful for the assessment of change of infection. Both WBC and CRP increased on the first day after surgery probably due to stress response. CRP and ESR decreased and reached normal range in about 1 week postoperatively. However, an irregular phenomenon was noticed on day 7, when all the parameters showed an increase after they gradually decreased. The reasons might be that the plots were the average value of each laboratory marker, which reported the central tendency. Thus, it is greatly influenced by outliers (values that are very much larger or smaller than most of the values).

OM occurring in the metaphysis in infants younger than 18 months old has a potential risk of physeal plate injury and growth disturbance [8, 10, 17]. Kao [18] reported three cases of physeal plate injury in his 12 years of clinical experience, which all required debridement for further treatment. Longjohn et al. [17] also reported poor outcome when OM concurred with septic arthritis. In the present study, patients were diagnosed with septic arthritis if a purulent material was found in the joint capsule during the operation. Other patients in whom pus was not found during the operation were diagnosed as isolated OM. Moreover, patients who had concurrent pyogenic arthritis were more likely to have complications than those with OM alone (*P* = 0.02). Therefore, once the diagnosis of OM is made, appropriate antibiotics should be started as soon as possible, because of the tendency for OM of metaphysis to develop secondary septic arthritis due to the unique structure of vessels in infants [19].

The treatment of OM often begins with an antibiotic intravenously. A more specific antibiotic should be chosen according to bacteria culture and drug sensitivity results as soon as possible [20]. Initial antibiotic choice should cover *Staphylococcus aureus* which is the most common pathogen [21, 22]. Subsequent administration of antibiotics should be adjusted according to the results of drug sensitivity test, clinical manifestations, and laboratory tests. Usually, 4-to 6-week course of antibiotics is required [18]. Nikolas A et al. [23] reported that only 50% of patients had positive blood culture in their 70-person study. In the current study, the overall positive rate of blood and pus culture was 31.82% (7/22). This might be associated with previous use of antibiotics prior to admission. Systemic administration of antibiotics may cause systemic side effects, such as diarrhea and liver function damage, as well as low concentration at the infection site, especially in infants. For these reasons, combining antibiotic therapy with surgical intervention is warranted.

OM without bone lesions has traditionally been treated with 4–6 weeks of antibiotics. In the current study, debridement surgery combined with RBGS was performed when a bone lesion was observed by radiological investigations in our institution. The choice of initial antibiotics was guided by local microbiology. Broad spectrum coverage was generally utilized in this population as infants may be infected with a wider variety of organisms. [24]

The use of a local delivery of antibiotics has advantages over traditional debridement because it increases the local concentration at the infection sites [25]. In previous animal studies, Penn-Barwell and Wencke [26] showed that local delivery of antibiotics into an infected bone defect was superior to systemic antibiotics alone at 14 days after surgery. Branstetter et al. [27] confirmed that calcium sulfate mixed with antibiotics eradicated bacteria better after debridement when compared with calcium sulfate alone. The use of RBGS mixed with vancomycin has been shown to be a successful outcome in the management of chronic osteomyelitis and non-union in adults [28, 29]. McNally MA et al. described effective results in the treatment of deep bone infection using a new antibiotic-loaded biocomposite in the eradication of infection from bone defects [30]. Also, a systematic review of 15 studies to access the utility of anti-infective bone graft substitutes in osteomyelitis treatment suggested that such treatment could be a good option [25]. Based on these reports, we were interested to determine if this treatment regimen would be effective in treating OM in infants.

**Fig. 2:**
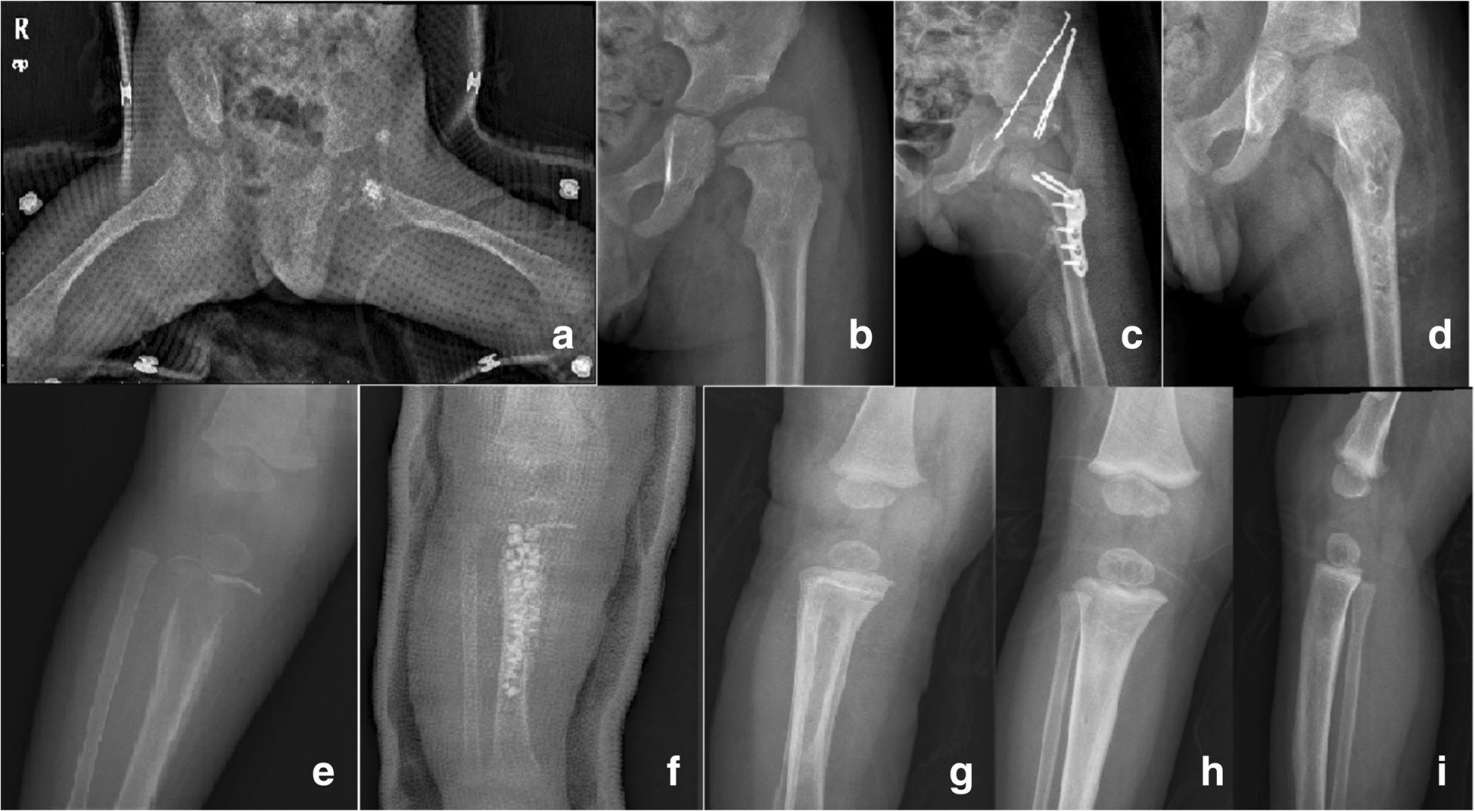
AHO with or without a secondary septic arthritis. **a** An 11-month-old boy, who suffered from osteomyelitis concurred with septic arthritis on the left proximal femur, had received debridement combined with RBGS mixed with antibiotics. The hip was immobilized with a brace postoperatively. **b** Pathological subluxation was observed after 3 years postoperatively. **c** An innominate osteotomy and varus derotational osteotomy of the proximal femur were performed to correct residual deformities **d** A limb shortening of 1 cm was noted at the final follow-up. **e** A 9-month-old boy who suffered from osteomyelitis on the right proximal tibia. **f** After being debrided, bone defect was filled with RBGS, which was mixed with 0.5 g vancomycin. The affected limb was immobilized by a cast postoperatively. **g** The bone lesion healed after 2 months postoperatively. **h**,**i** The outcome of the involved limb was good at the final follow-up

In the current study, RBGS mixed with antibiotics were implanted in the local defect after debridement. There are multiple benefits to this technique. The local delivery allows for a slow-release effect and can maintain a relatively higher antibiotic concentration locally. Local use of antibiotics has previously been shown to improve local concentrations. The use of tobramycin had local concentrations of about 1000 times above the minimal inhibitory concentration (MIC) for most strains of *Staphylococcus* [31]. Gentamicin-PMMA beads also showed high local concentrations, around 100 times above the MIC, and remained bactericidal several days later [32]. Local sustained availability of drugs is more effective in achieving prophylactic and therapeutic outcomes. It could also help to avoid high systemic levels of parenteral antibiotics which can lead to additional adverse effects, such as toxic liver injury and fungal infection [33]. Additionally, after debridement, a large defect usually occurred, which weakens the strength of the bone (Fig. 2). Filling the cavity with RBGS mixed with antibiotics can provide mechanical support as well as stimulate bone regrowth [34]. Furthermore, we stressed the importance of immobilization postoperatively to prevent pathological fractures.

Adult OM is commonly seen with contiguous infection caused by open fractures or joint replacement surgeries and vascular or neurologic insufficiency [35]. Antibiotic therapy is the first-choice treatment for acute OM, while debridement is indicated in chronic OM [36]. In infants, however, OM tends to occur in the site of the metaphysis, which has a potential risk of physeal plate injury. Based on that, aggressive surgical intervention is warranted to prevent the devastating complications such as LLD and AVN.

Recently, a new technology named supercritical emulsion extraction (SEE) had been confirmed to be a green, flexible way to make a multiloaded poly-lactic-co-glycolic acid (PLGA) microdevice for gentamicin (Gen) sustained release in an in vitro study. Gen molecules were not chemically changed by such technology and maintained their biological activity [37]. In the future, other new technologies may provide alternative treatment options by delivering in situ in a dose- and time-controlled manner pharmacologically active molecules to tackle bone infection.

There were some limitations in this study. Firstly, a small sample size decreased the power of our statistical analysis. However, this is the first study focused on infants with OM treated with RBGS mixed with antibiotics. Secondly, this retrospective study contained patients with different lesion sites which introduced bias into the results. Thirdly, lack of a control group of other treatment options and loss of follow-up also affected the statistical efficiency. At the final follow-up, we only observed seven patients with complications. Longer-term close follow-ups until skeletal maturity of those patients with good outcomes are necessary in our subsequent works. Finally, a multicenter and prospective study should be performed in the future to obtain more powerful evidence about this issue.

## Conclusions

We reported a single-stage protocol for treatment of infantile OM including early operative debridement and the use of RBGS mixed with vancomycin as a space filler. This protocol showed a low recurrence rate and few complications over one- to six-year follow-up period. These results suggest that this option in the treatment of OM might achieve acceptable results and merits further investigations.

## Abbreviations

AVN: Avascular necrosis
CRP: C-reactive protein
ESR: Erythrocyte sedimentation rate
Gen: Gentamicin
LLD: Limb length deformity
MIC: Minimal inhibitory concentration
NEU%: Neutrophil percentage
OM: Osteomyelitis
PLGA: Poly-lactic-co-glycolic acid
RBGS: Resorbable bone graft substitute
SEE: Supercritical emulsion extraction
WBC: White blood cell

## References

[1] Green NE, Edwards K (1987). Bone and joint infections in children. Orthop Clin North Am.

[2] El-Rosasy MA (2013). Ilizarov treatment for pseudarthrosis of the tibia due to haematogenous osteomyelitis. J Pediatr Orthop B.

[3] Matic A (2012). Acute osteomyelitis and septic arthritis of the shoulder in premature neonates--report of two cases. Med Pregl.

[4] Humm G (2014). Adjuvant treatment of chronic osteomyelitis of the tibia following exogenous trauma using OSTEOSET®-T: a review of 21 patients in a regional trauma Centre. Strategies Trauma Limb Reconstr.

[5] Fleiter N (2014). Clinical use and safety of a novel gentamicin-releasing resorbable bone graft substitute in the treatment of osteomyelitis/osteitis. Bone Joint Res.

[6] Agarwal A, Aggarwal AN (2016). Bone and joint infections in children: acute hematogenous osteomyelitis. Indian J Pediatr.

[7] Tuason DA (2014). Clinical and laboratory parameters associated with multiple surgeries in children with acute hematogenous osteomyelitis. J Pediatr Orthop.

[8] Rosenbaum DM, Blumhagen JD (1985). Acute epiphyseal osteomyelitis in children. Radiology.

[9] Kramer SJ, Post J, Sussman M (1986). Acute hematogenous osteomyelitis of the epiphysis. J Pediatr Orthop.

[10] Green NE, Beauchamp RD, Griffin PP (1981). Primary subacute epiphyseal osteomyelitis. J Bone Joint Surg Am.

[11] Montgomery CO (2013). Concurrent septic arthritis and osteomyelitis in children. J Pediatr Orthop.

[12] Porat S (1991). Complications of suppurative arthritis and osteomyelitis in children. Int Orthop.

[13] Goergens ED (2005). Acute osteomyelitis and septic arthritis in children. J Paediatr Child Health.

[14] Arnold JC (2012). Acute bacterial osteoarticular infections: eight-year analysis of C-reactive protein for oral step-down therapy. Pediatrics.

[15] Chou AC, Mahadev A (2016). The use of C-reactive protein as a guide for transitioning to oral antibiotics in pediatric osteoarticular infections. J Pediatr Orthop.

[16] Unkila-Kallio L, Kallio MJ, Peltola H (1994). The usefulness of C-reactive protein levels in the identification of concurrent septic arthritis in children who have acute hematogenous osteomyelitis. A comparison with the usefulness of the erythrocyte sedimentation rate and the white blood-cell count. J Bone Joint Surg Am.

[17] Longjohn DB, Zionts LE, Stott NS (1995). Acute hematogenous osteomyelitis of the epiphysis. Clin Orthop Relat Res.

[18] Kao FC (2003). Acute primary hematogenous osteomyelitis of the epiphysis: report of two cases. Chang Gung Med J.

[19] Bar-On E (2010). Chronic osteomyelitis in children: treatment by intramedullary reaming and antibiotic-impregnated cement rods. J Pediatr Orthop.

[20] Paakkonen M (2013). C-reactive protein versus erythrocyte sedimentation rate, white blood cell count and alkaline phosphatase in diagnosing bacteraemia in bone and joint infections. J Paediatr Child Health.

[21] Stone B (2016). Pediatric tibial osteomyelitis. J Pediatr Orthop.

[22] Belthur MV (2010). Prospective evaluation of a shortened regimen of treatment for acute osteomyelitis and septic arthritis in children. J Pediatr Orthop.

[23] Jagodzinski NA (2009). Prospective evaluation of a shortened regimen of treatment for acute osteomyelitis and septic arthritis in children. J Pediatr Orthop.

[24] Yeo A, Ramachandran M (2014). Acute haematogenous osteomyelitis in children. Bmj.

[25] van Vugt TA, Geurts J, Arts JJ (2016). Clinical application of antimicrobial bone graft substitute in osteomyelitis treatment: a systematic review of different bone graft substitutes available in clinical treatment of osteomyelitis. Biomed Res Int.

[26] Rand BC, Penn-Barwell JG, Wenke JC (2015). Combined local and systemic antibiotic delivery improves eradication of wound contamination: an animal experimental model of contaminated fracture. Bone Joint J.

[27] Branstetter JG (2009). Locally-administered antibiotics in wounds in a limb. J Bone Joint Surg Br.

[28] Vazquez M (2002). Osteomyelitis in children. Curr Opin Pediatr.

[29] Humm G (2014). Adjuvant treatment of chronic osteomyelitis of the tibia following exogenous trauma using OSTEOSET((R))-T: a review of 21 patients in a regional trauma Centre. Strategies Trauma Limb Reconstr.

[30] McNally MA (2016). Single-stage treatment of chronic osteomyelitis with a new absorbable, gentamicin-loaded, calcium sulphate/hydroxyapatite biocomposite: a prospective series of 100 cases. Bone Joint J.

[31] Wahl P (2011). Systemic exposure to tobramycin after local antibiotic treatment with calcium sulphate as carrier material. Arch Orthop Trauma Surg.

[32] Walenkamp GH, Vree TB, van Rens TJ (1986). Gentamicin-PMMA beads. Pharmacokinetic and nephrotoxicological study. Clin Orthop Relat Res.

[33] Zalavras CG, Patzakis MJ, Holtom P (2004). Local antibiotic therapy in the treatment of open fractures and osteomyelitis. Clin Orthop Relat Res.

[34] Canavese F (2017). Successful treatment of chronic osteomyelitis in children with debridement, antibiotic-laden cement spacer and bone graft substitute. Eur J Orthop Surg Traumatol.

[35] Waldvogel FA, Medoff G, Swartz MN (1970). Osteomyelitis: a review of clinical features, therapeutic considerations and unusual aspects. N Engl J Med.

[36] Maffulli N (2016). The management of osteomyelitis in the adult. Surgeon.

[37] Della Porta G (2016). Injectable PLGA/hydroxyapatite/chitosan microcapsules produced by supercritical emulsion extraction technology: an in vitro study on teriparatide/gentamicin controlled release. J Pharm Sci.

